# *ShadowOui*: a new visual environment for X-ray optics and synchrotron beamline simulations

**DOI:** 10.1107/S1600577516013837

**Published:** 2016-10-14

**Authors:** Luca Rebuffi, Manuel Sánchez del Río

**Affiliations:** a Elettra-Sincrotrone Trieste SCpA, Trieste, Italy; b European Synchrotron Radiation Facility, Grenoble, France

**Keywords:** *SHADOW*, *ShadowOui*, ray tracing, X-ray optics

## Abstract

A new computer environment to perform simulations on synchrotron experiments has been designed. It performs ray-tracing simulations using the popular ray-tracing code *SHADOW*. With this new application one can define, in a very easy and elegant way, one or several optical systems (beamlines) and perform calculations of the propagation of the X-ray beam through it. Many complementary tools and supplementary calculations improve and extend the functionality of *SHADOW* to deal with complex optical system optimization, including compound optical elements, iterative calculations, some sample simulations, and implementing corrections for wave optics *via* a hybrid model.

## Abstract 2

*ShadowOui* is introduced, a new computer environment for X-ray optics, beamline simulations and virtual experiments using the ray-tracing code *SHADOW*.

## 1. Introduction   

The design of any X-ray instrument, such as a synchrotron beamline, requires an accurate conceptual design of the optics. Software tools exist to simulate the behaviour of the optics of the instrument in the computer. For synchrotron radiation beamlines, these tools are developed and maintained by the synchrotron community, as most of the commercial available tools lack the functionality needed for synchrotron applications, like the simulation of X-ray sources (bending magnets, wigglers and undulators), the use of grazing-incidence optics, crystal optics, compound refractive lenses with high number of elements, and availability of the refraction indices and attenuation coefficients at X-ray wavelengths. The codes in use can be classified into two main groups: wave optics and ray tracing. Wave optics and physical optics are well adapted for the propagation of coherent waves and contain models that propagate wavefronts using typically Fresnel–Kirchhoff integrals with several approximations. On the other hand, ray tracing is mostly used for simulating and propagating in­coherent beams, by using a model that decomposes the beam into small monochromatic collimated beams (or rays): each ray is treated in the geometrical optical approximation, thus travelling along a straight line (solution of the Helmholtz equation when wavelength tends to zero). The photon beam is assumed to be formed by many rays added incoherently, by adding intensities instead of electric fields.

Some popular wave optics codes for synchrotron radiation are *SRW* (Chubar & Elleaume, 1998 ▸) and *PHASE* (Bahrdt *et al.*, 2011 ▸). Other wave optics packages can be used for the propagation and of a coherent wavefront and its interaction with objects or optical elements [*e.g.* *XWFP* (Weitkamp, 2004 ▸)]. Ray-tracing codes have been used for the design and optimization of beamlines at most synchrotrons. Although new packages have been proposed in recent years [*McXtrace* (Knudsen *et al.*, 2011 ▸), *XRT* (Klementiev & Chernikov, 2014 ▸)], the code *SHADOW* (Cerrina & Sánchez del Rio, 2010 ▸) created by Professor Cerrina in the 1980s remains the most popular and widely used tools because it is specifically geared to the synchrotron radiation domain, it is flexible to calculate different beamline configurations, as well as it having a long list of publications. The refactoring and modernization introduced in the last *SHADOW3* (Sanchez del Rio *et al.*, 2011 ▸) version guarantees its easy installation, compilation, maintenance and use in any modern computer environment. It is relatively simple to use, documented, includes a Python application programming interface (API), and is freely available (open source).

*SHADOW* is based on a geometrical ray-tracing approach, but also traces field amplitude with phase difference, and therefore is capable of including reflectivity and transmittance of optical elements calculated by models of physical optics. It may also incorporate wave features beyond the validity domain of geometric optics. In recent years we attended to a tremendous growth of techniques exploiting the coherence of the synchrotron beam (phase contrast imaging, coherent diffraction imaging, ptychography, *etc*.) and this is also supported by new techniques exploiting the total coherence of X-FEL sources as well as in the much improved coherence of the synchrotron beams in the new storage rings. These machines are designed to reduce the horizontal emittance to values comparable with the present vertical emittances, like the EBS, the new planned storage ring at the ESRF (Admans *et al.*, 2014 ▸). Without dismissing the interest of these trends, these facts pushed some to believe that only issues related to physical optics and propagation of coherent beams are interesting. It is thus important to remark that the synchrotron beam from a storage beam is partially coherent, thus not fully coherent nor incoherent. The rigorous treatment of the partial coherence in synchrotron beams is not yet implemented in any available simulation package. There are, however, many efforts to approach partial coherence from fully coherence methods, like the multi-electron calculations of *SRW*. On the other hand, the Hybrid method (Shi *et al.*, 2014 ▸) corrects the ray-tracing results of *SHADOW* with effects due to coherence diffraction and propagation. To illustrate how simulations with incoherent optics are important one can think that, even for the EBS, the coherent fraction or undulator light emitted at about 20 keV is around 1%, thus 99% of the emitted photons can be considered incoherent.

In this work we present *ShadowOui*, a completely new visual environment for X-ray optics, beamline simulations and virtual experiments that uses *SHADOW3* as calculation engine for ray-tracing simulations. It also includes new tools and techniques, and is embedded in an environment called *OASYS* [*OrAnge SYnchrotron Suite* (Sánchez del Rio *et al.*, 2014 ▸)], that allows interfacing other new and existing codes and communicating with them.

## 2. Design of a modern simulation environment for synchrotron optics   

After the refactoring of *SHADOW* and the release of *SHADOW3* in 2011, it was required to renew the old graphics tools and interfaces, based on *XOP* (Sánchez del Río & Dejus, 2011 ▸). Our experience showed that the efficiency of *SHADOW* is supported by a well designed and user-friendly user interface. For about 20 years, more than 90% of the *SHADOW* calculations used the *ShadowVUI* interface available in *XOP*. Evolution of the hardware platforms, modernization of the software tools, access to the codes of a large number of young people and popularization of the open source software for scientific applications drove us to design a completely new graphical user interface for *SHADOW3*. The interaction with a large community of users permitted us to identify a list of requirements:

(i) The interface should be easy and intuitive to use.

(ii) The interface should make use of existing, state-of-the art and very well tested calculation engines.

(iii) Allow high flexibility and rapidity for interactive simulation and changes in beamline configurations.

(iv) Save and reuse systems (workspaces).

(v) Possibility to compare different beamlines and switch different optics: the workspace could contain several beamlines and configurations.

(vi) Portability: designed to be used in personal computers, in particular laptops.

(vii) Extensibility: possibility to integrate and communicate with other large simulation tools for optics (*e.g.* wave optics). It should also be extensible and integrate software for simulations before the photon source (*e.g.* accelerator optics) and after the sample (analysers and sample simulations).

Some solutions have been chosen:

(i) Use Python as the main programming language, because of its universality and popularity in scientific computing. It is a very concise language with numerous libraries available.

(ii) A high-level interface based on workflows. We selected *Orange* (Orange, 2016 ▸), which was customized into *OASYS*, our development platform.

(iii) *SHADOW3* was selected as the main calculation engine, because it is the most-used ray-tracing code in the community, and it has a Python API. *SHADOW3* is used only *via* the simplified API interface, meaning that no system calls are performed as done in previous graphical interfaces for *SHADOW* which communicate only *via* files. All previous calculations required by *SHADOW* (*e.g.* the pre-processors for preparing the physical properties of materials) have been completely rewritten in Python, using the xraylib library (Schoonjans *et al.*, 2011 ▸). Similarly, all the new post-processors are fully implemented in Python.

(iv) Graphics (one-dimensional, two-dimensional, histograms, *etc*.) are presently using *PyMca* (Solé *et al.*, 2007 ▸) graphics. They will be soon replaced by *silx* (Silx, 2016 ▸).

(v) Widgets use the *Qt* library, *via* the pyqt binding. This is also the standard in *Orange* and *silx*.

**Figure 1:**
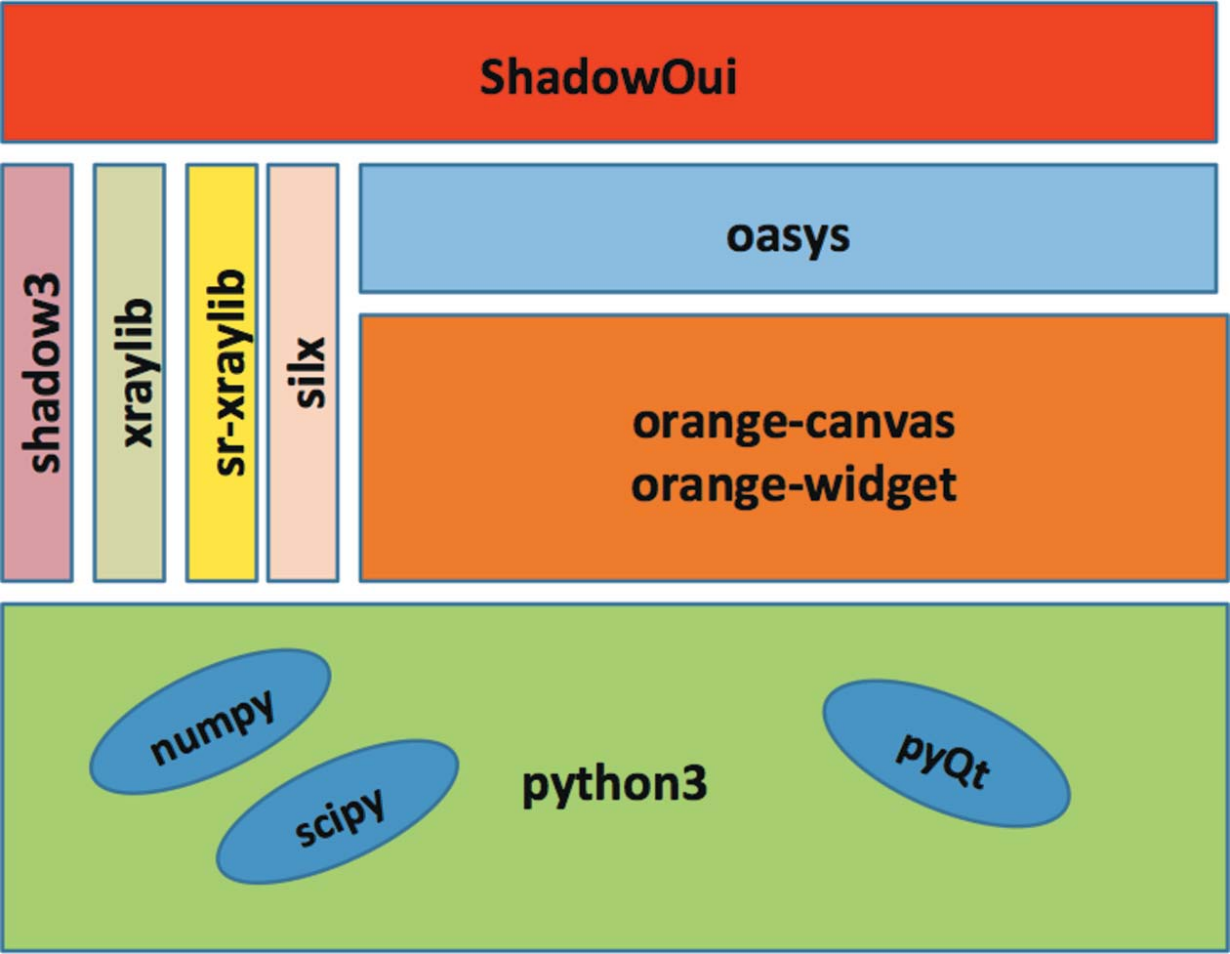
Block diagram of *ShadowOui*.

A block diagram of the modules that constitute *ShadowOui* is shown in Fig. 1 ▸.

## 3. Main use of *ShadowOui*   

**Figure 2:**
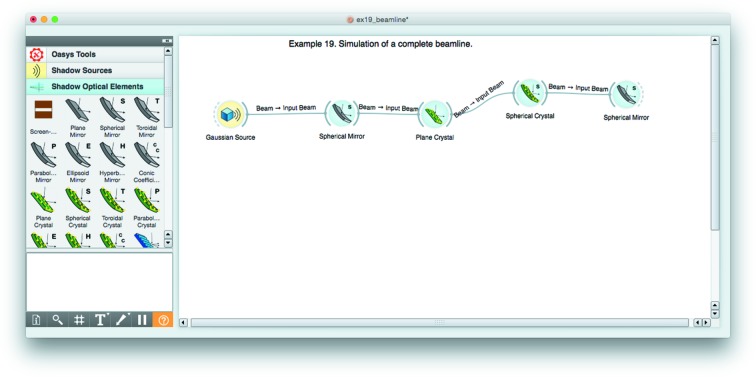
The *ShadowOui* application showing the *OASYS* canvas, the *ShadowOui* menus and an example beamline.

From the user point of view, the *ShadowOui* package is presented with several parts (see Fig. 2 ▸):

(i) Canvas: this is the space for working, to be populated with different elements (called widgets).

(ii) ToolBox: the menu with the items that are used to populate the canvas with elements from different categories: sources, optical elements and accessory tools, *etc*.

(iii) Connectors: the wires that connect widgets building the schema. The information (the beam, containing the rays and their history) travels through these wires.

(iv) Widgets: the active elements. Double-clicking in a widget opens a window, containing the parameters to be defined and customized.

An action ‘run’ must be started from source widgets. The signal (X-ray beam) is propagated automatically through all connected elements. The results are stored in computer memory. A change of parameters in any widget and ‘run’ in that widget implies re-running all elements downstream of it. The information after running is available for any widget inside the popped up parameters window. Other widgets (Info, Plot XY, Histogram, *etc*.) can be connected for a more precise visualization and analysis.

The provided solution presents a number of advantages:

(i) An optimum and intuitive view of the system that is implemented.

(ii) High modularity: each component deals with its own parameters and shows the local graphics or results, depending on where it is positioned.

(iii) *Orange* provides a mechanism to make annotations in the canvas, to maintain the parameters of each widget updated and to save and reload workspaces in files. It is very simple to restore workspaces (beamline simulations) and to exchange them among different users or designers.

The user familiarized with *SHADOW* will find some similarities in the menu parameters found in different components (widgets). However, after a complete study considering the feedback from many users of the old *SHADOW* interfaces over many years, some aspects have been redesigned in order to make life easier for the user:

(i) *SHADOW* uses internally a unique optical element (made by a single optical surface) that is customized *via* its internal parameters into a different concept of element selecting the adequate geometry (plane, bent cylindrical, ellipsoidal, *etc*.) and how to scatter the beam from the physical point of view (mirror, grating or crystal). This is not very intuitive for users, where the concepts, for example, of being a mirror or being a crystal are completely different. Therefore, we evolved from the idea of a single optical element into multiple optical elements implemented in multiple widgets. We have now three physical elements (mirrors, gratings and crystals) with seven possible geometries (plane, sphere, toroid, paraboloid, ellipsoid, hyperboloid, conic coefficients).

(ii) In *SHADOW*, an aperture (slit), a beam stopper (‘negative slit’), a simple monitor plane (screen) or an absorber (filter or attenuator) enters into the same family concept ‘screen’. They are not identified as ‘optical elements’ but presented associated with existing optical elements. We decided in *ShadowOui* to promote ‘screens’ to the level of ‘optical element’ and define them using an independent ‘screen widget’.

(iii) Two more elements available in *SHADOW* are also presented as widgets: the refractor interface and the empty element. The latter is useful for changing the orientation of the optical axes without altering the beam evolution.

(iv) *ShadowOui* offers to the user a new family of elements not existing as individual elements in *SHADOW* but built using *SHADOW* components. These are the ‘compound optical elements’. Typical cases are the ‘double-crystal monochromator’ and the ‘Kirkpatrick–Baez system’ where the user defines now in an easier way the parameters in a single widget instead of defining two separate widgets. The use of compound optical elements is fully exploited for lenses. The *ShadowOui* compound elements Lens, Compound Refractive Lens and Transfocator facilitate the definition of these elements.

(v) The Info widget centralizes all the information obtained in the old *SHADOW* by running individual post-processors. It displays information of the source (sourcinfo in the original *SHADOW*), the individual optical elements (mirinfo), the grouped elements in the optical system (sysinfo) and a summary of distances between elements. It also supplies a Python script that implements the current calculation. This is a very powerful tool that can be used as a starting point of advanced optimization by user code.

## 4. Advanced simulation tools   

As mentioned before, *ShadowOui* not only implements the functionality of *SHADOW3* but it highly improves the ways to define the optical system and incorporates new tools that make easier, for example, the definition of the slope errors, to include X-ray lenses in the beamline, or to study the effect of beam coherence in the beamline. These new tools are described in the following paragraphs.

### 4.1. Loops   

**Figure 3:**
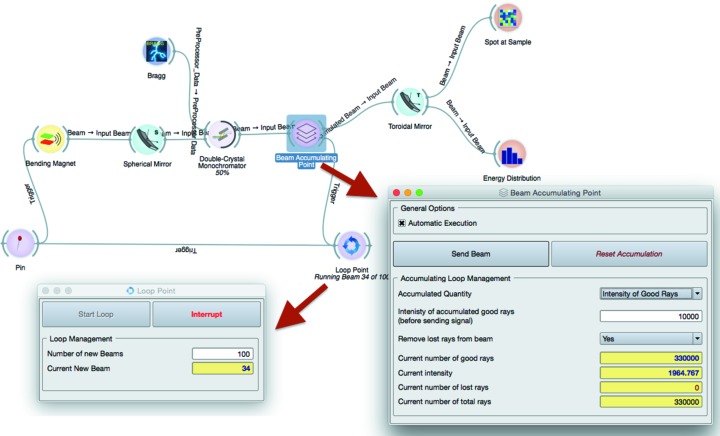
Implementation of a loop in *ShadowOui* using the Loop Point widget and Beam Accumulating Point widget.

A very useful feature of *SHADOW* is that it traces all rays through the sequential elements of the beamline, allowing at any time after the run to visualize *a posteriori* whatever parameter we are interested in [*e.g.* cross section (*x*, *z*), horizontal phase space (*x*, *x*′)]. However, storing all rays in memory makes it impossible to run a very large amount of rays. Typically a single run can have 10^3^ to 10^6^ rays, and if the user needs more rays a loop accumulating results of different runs must be implemented. A special feature of recursive and cumulative simulations has been introduced. It improves the statistical quality of the simulation, avoiding the use of a single run with a huge amount of generated rays, but executing a series of several simulations with a small number of generated rays. It has the double advantage of monitoring the simulation during its execution and reducing the memory allocation (see Fig. 3 ▸). This feature is particularly useful where the execution time of the algorithm is not linear with the number of rays, like in the simulation of samples.

Another looping tool is the Beam Accumulation Point widget (see Fig. 3 ▸), with the function of accumulating good rays during a loop until a desired amount of rays or a specific intensity (sum over the rays of the squared modulus of the electric field) of the beam is reached, then sending the accumulated beam to further elements. This widget is particularly useful when the reflectivity of crystals and mirrors are taken into account, potentially reducing the intensity of the beam to small numbers, despite a statistically relevant amount of good rays in the beam.

### 4.2. Optical surface errors   

*SHADOW3* already contained the Waviness algorithm able to add a surface error profile to geometrically perfect surfaces of optical elements like mirrors, generating a two-dimensional mesh of the surface error profile to be added to the optical element surface, by adding a certain amount of sinusoidal harmonics (Sánchez del Río & Marcelli, 1992 ▸), whose amplitude is modulated by the desired estimated slope error. This functionality has been imported into *ShadowOui*, together with its visualization tools, in order to allow the user to immediately check the goodness of the generated surface error profile.

**Figure 4:**
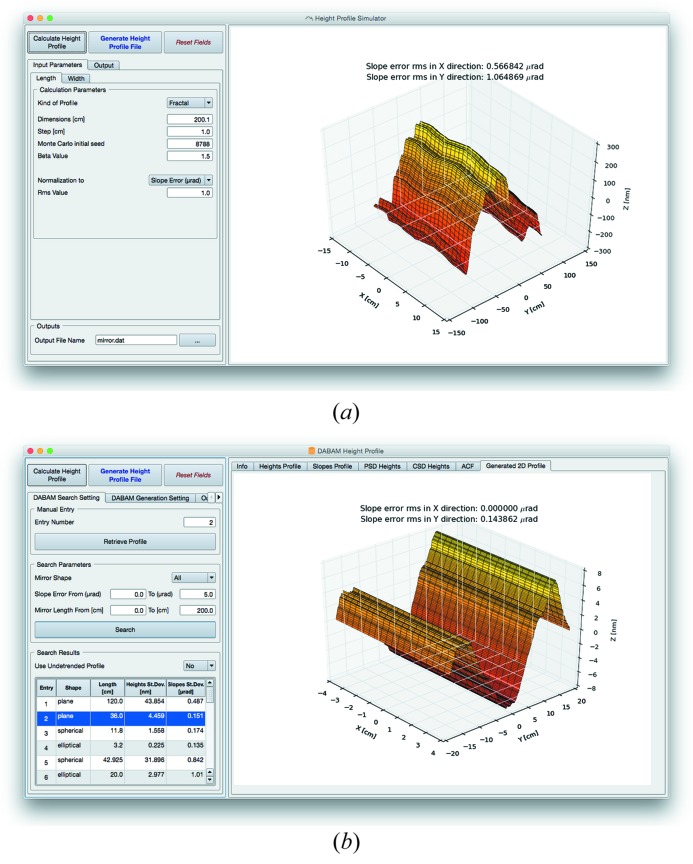
Input/output form of the Height Error Profile (*a*) and DABAM widgets (*b*): the calculated surface mesh is rendered to be checked by the user.

*ShadowOui* also contains more evolved tools, *i.e.* the Height Profile Simulator widget (see Fig. 4*a* ▸), capable of separately generating the error profile along the longitudinal and transversal directions, with different values of slope or figure error. The two-dimensional mesh of the surface error profile to be added to the surface of the optical element, with the same format of the Waviness algorithm, can be generated using different algorithms: fractal or Gaussian distribution of errors, and also from a user-defined error profile from an external file, thus allowing the use of experimental or customized error profiles.

Finally, the user can generate the two-dimensional mesh of the error profile using the DABAM Height Profile widget (see Fig. 4*b* ▸), accessing in an automatic and transparent way the DABAM online metrology database (Sanchez del Rio *et al.*, 2016 ▸), containing a collection of experimental error profiles, coming from a worldwide group of metrology laboratories. With the DABAM widget the user can analyse the behaviour of a beamline by simulating mirrors with experimental error profiles contained in the database, querying the database with geometrical parameters and manipulating the chosen profile in order to properly fit the simulated optical element, both in terms of dimension and in terms of final slope errors.

### 4.3. Compound optical elements   

*ShadowOui* contains a new class of optical elements that combine single elements into a compound one. Even though *SHADOW* will always see each element individually, the interface presents them to the user as a single element facilitating the use. The Double-crystal monochromator allows the most used beamline monochromator in a single widget to be defined. In a similar way, the Kirkpatrick–Baez KB) widget allows to easily define the parameters of this popular mirror configuration, defining, for example, the mirror distances related to the center of the KB. The use of compound elements is fundamental for the X-ray lens systems, like the Single lens (made by two lens interfaces), the Compound Refractive Lens (made by *N* identical lenses) and the Transfocator (made by *M* different compound refractive lenses). Before *ShadowOui*, it was almost impossible to use compound lenses without using cumbersome scripts to define the parameters of the lenses. Moreover, the fact that *ShadowOui* by default does not dump disk files makes the *SHADOW* calculation fast enough even for hundreds of optical elements.

### 4.4. Hybrid method   

The so-called Hybrid method computes diffraction effects when the beam is clipped by an aperture or mirror length and can also simulate the effect of figure errors in the optical elements when diffraction is present (Shi *et al.*, 2014 ▸). This method has been implemented into *ShadowOui* as a dedicated widget, redesigning the user interface with a high level of automation.

**Figure 5:**
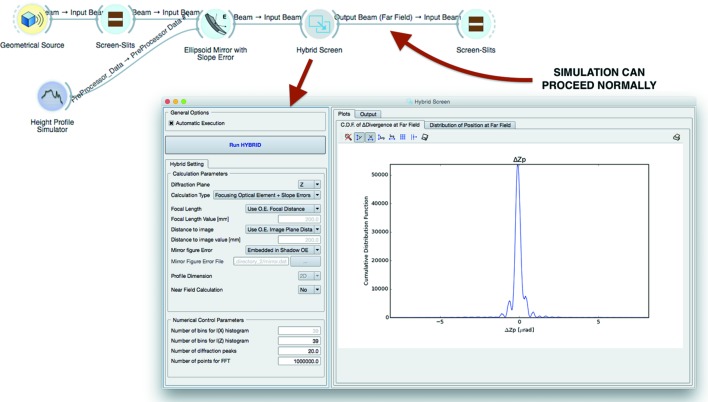
Hybrid widget: position in the beamline layout and input/output form with an example result.

**Figure 6:**
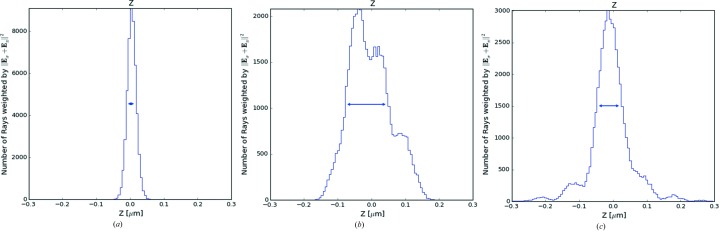
Comparison of simulation results, intensity *versus* *Z* coordinate plot at the mirror focus image plane with three different setups: ellipsoid mirror without error profile calculated (*a*), ellipsoid mirror with error profile (*b*), ellipsoid mirror with error profile calculated by Hybrid (*c*).

The Hybrid Screen widget supports three calculation types: (i) Simple Aperture, (ii) Focusing Optical Element, (iii) Focusing Optical Element + Slope Errors, and it is inserted between two optical elements, following the optical element to which the calculation is referred. The widget can extract all the needed information automatically from the input beam, generating, after the calculation, the output beam to be sent to further optical elements or to plot widgets, proceeding with the simulation normally, as visible in Fig. 5 ▸. Even in this case, all the relevant plots are automatically presented to the user after the calculation in the input/output form of the widget. Fig. 5 ▸ also shows the *ShadowOui* representation of an example of the last calculation type, the same as described in the reference literature (Shi *et al.*, 2014 ▸). In the new environment a two-dimensional height error profile is attached to the mirror, generated by the Height Error Profile widget, and automatically used by the Hybrid widget following the mirror. From this simulation layout, it was possible to produce three different results: the pure ray-tracing simulation of the optical system with and without the error profile on the mirror, and the Hybrid method calculation result, observing the effects of the interference of the beam with the error profile (see Fig. 6 ▸).

The Hybrid method is a fast algorithm able to produce results using a CPU time compatible with a typical *SHADOW* ray-tracing simulation, and it is accurate enough to be useful for beamline design purposes. Thanks to the *OASYS* engine, its usage became not only fully integrated into *ShadowOui* but more intuitive and user-friendly.

### 4.5. Simulation of samples   

Most X-ray techniques are based on a signal determined by the convolution of physical effects introduced by the sample with the characteristics of the photon beam mainly due to the source and beamline optics. The latter is the so-called Instrumental Profile Function (Cheary *et al.*, 2004 ▸; Zuev, 2006 ▸).

The ray-tracing approach can be used to *ab initio* calculate the instrumental function of a synchrotron radiation beamline. This has been successfully tested with an X-ray powder diffraction (XRPD) beamline (Rebuffi & Scardi, 2014 ▸) used for line profile analysis, where complete control of the diffracted signal is necessary (Scardi *et al.*, 2010 ▸). The synchrotron beam shape, divergence and energy distributions that result from the source characteristics and beamline optics contribute to broaden the diffraction peaks of the recorded diffractograms. The peak width dependence *versus* the 2θ angle (Caglioti *et al.*, 1958 ▸; Sabine, 1987 ▸) is usually parameterized by Caglioti’s equation (Scardi *et al.*, 1994 ▸), where the full width at half-maximum (FWHM) of the instrumental peak profiles represented as pseudo-Voigt curves has the form FWHM(θ) = [*W* + *V*tanθ + *U*tan^2^θ]^1/2^, where *W*, *V* and *U* are Caglioti’s parameters and θ is the diffraction angle.

**Figure 7:**
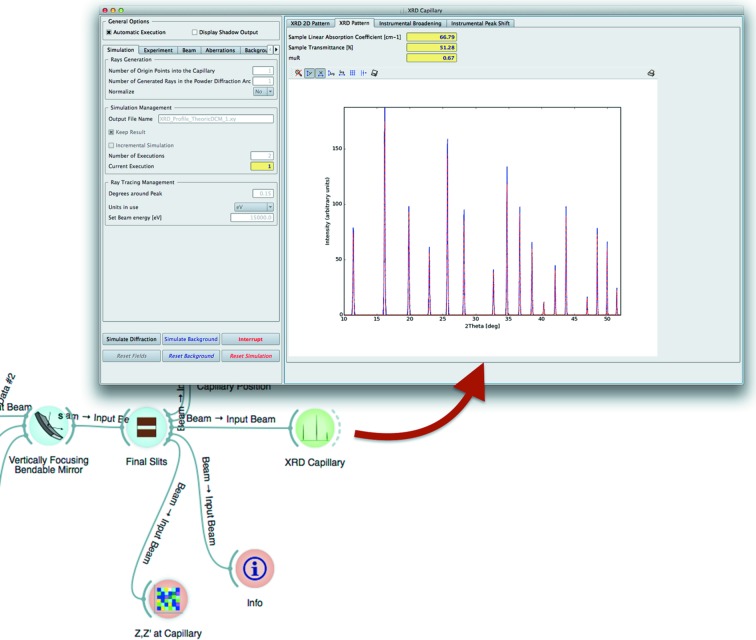
XRPD instrumental profile widget: position in the beamline layout and calculation example in the input/output form.

A special widget representing XRPD samples (see Fig. 7 ▸) in a capillary holder simulates the diffracted photon beam created by the interaction of the photon beam generated by *SHADOW* with a capillary filled by a crystalline material. The simulation takes into account not only the diffraction law but also the absorption of the photons by the sample material and the sample holder (that can be a source of considerable aberrations). Then, diffracted rays are traced onto the detector and other possible optical elements. The widget computes Caglioti’s parameters fitting a pseudo-Voigt at every simulated diffraction peak.

Every source of aberration coming from the beamline and the detecting system (diffractometer and detector in this case) are naturally taken into account by the ray-tracing procedure, without the need of introducing *ad hoc* models. Therefore, effects affecting the shape and width of the diffraction peaks introduced by the beamline optics like height error profiles in mirrors, crystal monochromator reflectivities and optical elements misalignments are *ab initio* simulated by *ShadowOui*.

The emerging paradigm is that models for beam–sample interaction can be introduced after the optics calculation for evaluating the instrument effect in the recorded experimental data. This may help not only to evaluate, tune and optimize the beamline parameters but also to compute instrumental functions that can be used in the data analysis. It can be certainly applied not only in diffraction but also in many X-ray techniques such as spectroscopy and imaging. The availability of our code in open source makes possible for interested users and developers to add their own models for photon–sample interaction and contribute with new widgets to the *OASYS*–*ShadowOui* project.

## 5. Examples   

### 5.1. Soft X-ray beamline   

**Figure 8:**
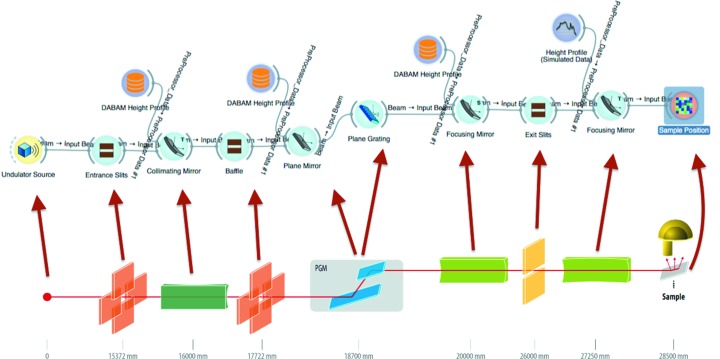
Optical layout of the UARPES beamline and its representation in *ShadowOui*.

As an example of a soft X-ray beamline, we simulate UARPES (angle-resolved photoelectron spectroscopy) at the Polish synchrotron Solaris (Szamota-Leandersson, 2016 ▸). The parameters for the simulations are reported in Table 1 ▸, and the schematic setup of the beamline is represented in Fig. 8 ▸, together with the *ShadowOui* schema.

For this example, experimental metrological data of all the four mirrors are available. Three complete error profiles have been submitted to the DABAM database (profiles numbers 27–29) and used in the simulation using the DABAM widget, while for the last a simulated profile with 1.5 µrad slope error is created using the Height Error Profile widget.

**Figure 9:**
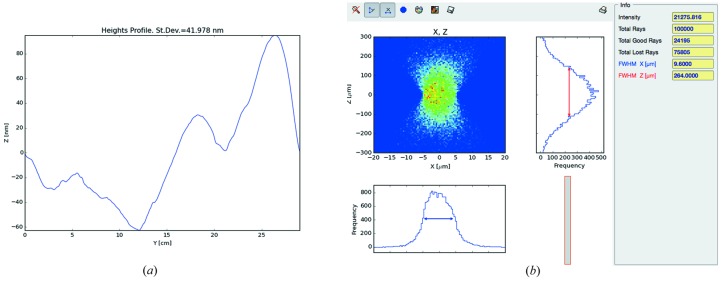
DABAM Heights Profile of the collimating mirror (*a*) and spot in an image plane corresponding to the sample position (*b*).

In Fig. 9 ▸ it is possible to see an example of one of the used DABAM heights profiles and the bidimensional plot of the intensity *versus* *X–Z* coordinates (spot) of the rays in the image plane, given by the Plot XY widget and corresponding to the sample position.

### 5.2. Hard X-ray beamline   

In this example the simulation of a typical hard X-ray beamline is shown, the XRD1 protein crystallography beamline at Elettra-Sincrotrone Trieste (Polentarutti, 2016 ▸).

**Figure 10:**
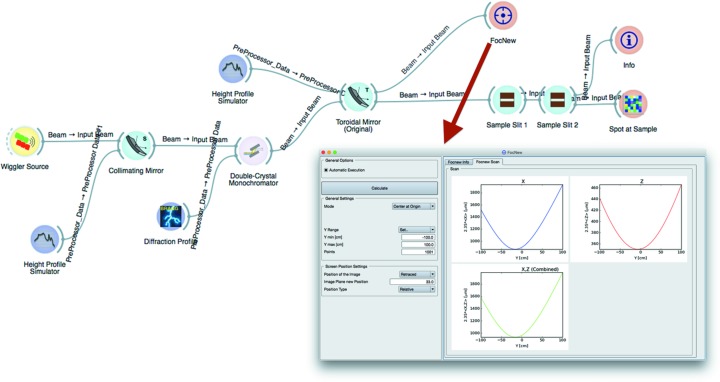
Complete simulation layout and FocNew widget input/output form with an example result: focus location of the XRD1 toroidal focusing mirror.

Technical details are reported in Table 2 ▸, and Fig. 10 ▸ shows the complete layout of the beamline, as implemented in *ShadowOui*.

**Figure 11:**
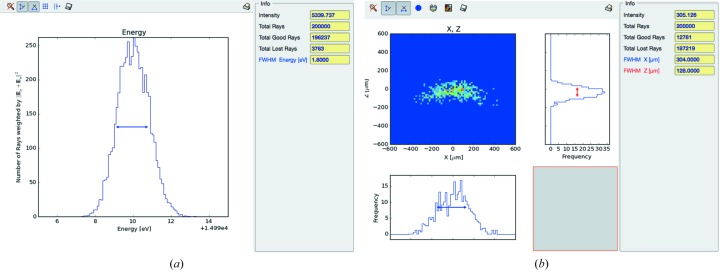
XRD1 beamline simulation results: intensity *versus* energy plot (*a*) and spot at the sample position (*b*).

The Wiggler Source widget is optimized to generate rays within angular acceptance of the front-end mask. The Height Profile Simulator widget is used to generate the surface errors, according to the measured slope error of 1.0 µrad and 1.5 µrad r.m.s. for the first collimating and the second focusing mirror, respectively. The double-crystal monochromator is represented by the dedicated Compound Optical Element widget. The final system of slits, shaping the beam in proximity of the sample of the experiment, is represented by two Screen-Slits widgets, configured as rectangular apertures. Other widgets complete the simulation: plotting tools (histogram of the energy distribution emerging from the monochromator and bidimensional plots of the *X–Z* coordinates of the rays in the image plane), the Info widget at the end of the beamline (containing the *SHADOW* functions SysInfo, MirInfo, SourceInfo and Distances) and the FocNew widget that computes the position of the best focuses visible in Fig. 10 ▸. In Fig. 11 ▸ a histogram of the energy distribution emerging from the monochromator and the spot shape at the sample position are shown.

### 5.3. Wiggler sources   

**Figure 12:**
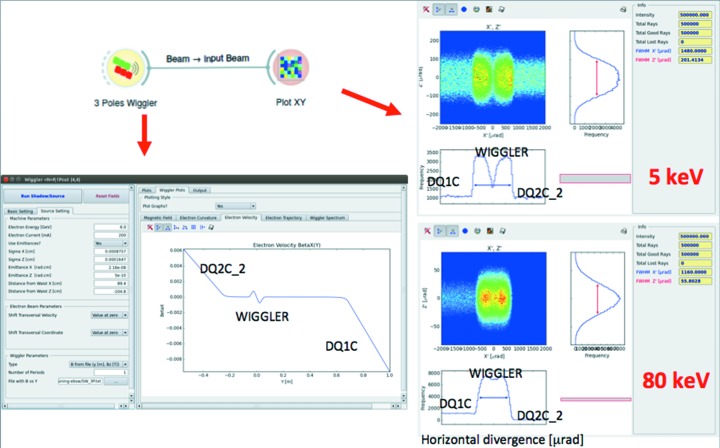
Simulation of a three-pole wiggler inserted into two bending magnets. Left: plot of the electron velocity showing the effect of the short wiggler in between the two dipoles. Right: intensity plot in divergence space for emission at 5 and 80 keV.

The ‘Wiggler’ widget in *ShadowOui* has been upgraded to calculate the emission from a wiggler placed in between other bending magnets. This was used for simulating the new ‘bending magnet’ beamlines for the future EBS source at the ESRF using the hybrid multi-bend achromat lattice. This lattice replaces the old intense bending magnets (magnetic field *B* ≃ 0.8 T, critical energy *E*_c_ ≃ 20 keV) by several shorter but less intense dipoles (*B* ≃ 0.4, 0.6 T, *E*_c_ ≃ 10, 15 keV, respectively). To keep these beamlines performant, the solution of placing a short insertion device in a narrow (10–15 cm) space left in between other magnets is proposed (Admans *et al.*, 2014 ▸). Different solutions have been studied for short wigglers with three or less poles. The proximity of the bending magnets introduces an overlapping of the radiation of the wiggler with the emission of the bending magnets. The simulation of the wiggler source together with the dipole sources is essential for selecting the best wiggler configuration reducing the overlapping as well as optimizing the divergence and geometry of the emitted and focused beams. The Wiggler widget in *ShadowOui* accepts a map of the magnetic field from which it calculates the electron velocities and trajectories by integration. The correct selection of the initial electron velocities and positions is essential to correctly align the optical axis of the beamline with the wiggler emission. These parameters can now be entered and modified by the user. The Wiggler widget displays the magnetic field, velocity, trajectory and photon energy spectrum, as well as the distribution of the source rays. For example, Fig. 12 ▸ shows the emission of a three-pole wiggler, showing a hole in the radiation at the centre of the emission because of the trajectory geometry. This figure also shows the overlapping and different effect of the side dipoles for low (5 keV) and high (80 keV) photon energies.

## 6. Summary and future work   

We have presented a new graphical application *ShadowOui* that allows optical systems to be simulated using the *SHADOW* engine. This tool has been designed to help the user to make simulations in a very intuitive and simple way. The power of *SHADOW* is complemented and extended with other tools that make it possible, for instance, to quickly integrate slope errors in mirrors or estimate the effect of coherent diffraction in the simulations.

The *ShadowOui* tool has been designed with the idea of being combined with other packages, for example, for quick calculations of characteristics of optical elements (like *XOP*) or for wave optics simulations. These tools will be developed in the near future, and integrated into the main *OASYS* platform. *OASYS* is now using *ShadowOui* as its only package (add-on) but accepts other packages that will be presented to the user in the same environment, allowing simulating beamlines with different tools without multiplying the beamline definitions.

All these software packages are created by and targeted at the synchrotron community and provided in open source. We welcome and encourage collaboration with individuals and institutions to maintain and develop these tools, and to interface and integrate other codes and packages in the *OASYS* environment. All necessary information for downloading and installing plus documentation, tutorials and examples (including workspaces for the examples in this paper) can be accessed from http://www.elettra.eu/oasys.html.

## Figures and Tables

**Table 1:** Optical layout of the UARPES beamline at Solaris synchrotron

Type	Description	Distance from source (m)
Source	APPLE-II type undulator (Sasaki, 1994 ▸)	
Slits	Horizontal and vertical slits	15.372
Mirror	Vertically collimating, horizontally focusing, Au-coated, toroidal mirror	16.000
Slits	Horizontal and vertical slits	17.772
Monochromator	Plane, Au-coated mirror and 600 lines mm^−1^ Au grating	18.700
Mirror	Cylindrical, Au-coated mirror	20.000
Slits	Vertical slits	26.000
Mirror	Vertically and horizontally focusing, Au-coated toroidal mirror	27.250
Sample holder	Spot size of 250 µm × 10 µm	28.500

**Table 2:** Optical layout of XRD1 beamline at Elettra synchrotron

Type	Description	Distance from source (m)
Wiggler source	Hybrid multipole wiggler (Bernstorff *et al.*, 1995 ▸)	
Mask	Front-end angular acceptance: 1.5 mrad × 0.182 mrad	10.000
Filters	Cooled graphite layers, with cut-off energy = 4 keV	13.000
Mirror	Vertically collimating, Pt-coated, cylindrical (tangentially bendable) mirror.	22.300
Monochromator	Nitrogen-cooled Si(111) double-crystal monochromator	24.500
Mirror	Vertically and horizontally focusing, Pt-coated toroidal (tangentially bendable) mirror	28.000
Slits 1	Vertical and horizontal slits	37.800
Slits 2	Vertical and horizontal slits	38.700
Sample position	Spot size of 0.7 mm × 0.2 mm	41.000
